# The OGF-OGFr axis utilizes the p21 pathway to restrict progression of human pancreatic cancer

**DOI:** 10.1186/1476-4598-7-5

**Published:** 2008-01-11

**Authors:** Fan Cheng, Patricia J McLaughlin, Michael F Verderame, Ian S Zagon

**Affiliations:** 1 Department of Neural and Behavioral Sciences, The Pennsylvania State University College of Medicine, Hershey, PA, USA; 2 Department of Medicine, The Pennsylvania State University College of Medicine, Hershey, PA, USA

## Abstract

**Background:**

Pancreatic cancer is the 4th leading cause of death from cancer in the U.S. The opioid growth factor (OGF; [Met^5^]-enkephalin) and the OGF receptor form an inhibitory growth regulatory system involved in the pathogenesis and treatment of pancreatic cancer. The OGF-OGFr axis influences the G_0_/G_1 _phase of the cell cycle. In this investigation, we elucidate the pathway of OGF in the cell cycle.

**Results:**

Using BxPC-3 cells, OGF decreased phosphorylation of retinoblastoma (Rb) protein without changing total Rb. This change was correlated with reduced cyclin-dependent kinase protein (Cdk) 2 kinase activity, but not total Cdk2. OGF treatment increased cyclin-dependent kinase inhibitor (CKI) p21 protein expression in comparison to controls, as well levels of p21 complexed with Cdk2. Naloxone abolished the increased expression of p21 protein by OGF, suggesting a receptor-mediated activity. p21 specific siRNAs blocked OGF's repressive action on proliferation in BxPC-3, PANC-1, and Capan-2 cells; cells transfected with negative control siRNA had no alteration in p21 expression, and therefore were inhibited by OGF.

**Conclusion:**

These data are the first to reveal that the target of cell proliferative inhibitory action of OGF in human pancreatic cancer is a p21 CKI pathway, expanding strategies for diagnosis and treatment of these neoplasias.

## Background

The opioid growth factor (OGF), chemically termed [Met^5^]-enkephalin, is an endogenous opioid peptide that is an important regulator of the progression of human pancreatic cancer [1-3]. OGF is a constitutively expressed native opioid that is autocrine produced and secreted, and interacts with the OGF receptor (OGFr) to inhibit the growth of pancreatic cancer cells *in vitro *and in tumor xenografts [2,3]. The action of OGF is tonic, stereospecific, reversible, non-cytotoxic and non-apoptotic inducing, not associated with differentiative, migratory, invasive, or adhesive processes, independent of serum, anchorage-independent, and occurs at physiologically relevant concentrations in a wide variety of pancreatic cancers including poorly- and well-differentiated human cell lines [1-3]. The only opioid peptide, natural or synthetic, that influences the growth of pancreatic cancer is OGF [2]. The action of this opioid in these neoplasias is targeted to DNA synthesis [4] and is directed toward the G_0_/G_1 _interface of the cell cycle [5]. Exogenous administration of OGF has a profound antitumor action on xenografts of pancreatic cancer that includes delaying tumor appearance and reducing tumor size [6]. The combination of biotherapy with OGF and chemotherapy with gemcitabine has proven to enhance antitumor effectiveness beyond either agent alone [6]. OGF has been successful in Phase 1 clinical trials [7].

The gene for human OGFr is at least 9 kb in length, consists of seven exons and six introns, and encodes a 677 amino acid protein that includes 7 imperfect repeats of 20 amino acids each and a bipartite nuclear localization signal [1]. OGFr has an apparent mass of 62 kDa. The chromosomal location of the human OGFr is 20q13.3 [1]. Although OGFr has characteristics of a classical opioid receptor (recognizes opioids, naloxone reversibility, stereospecificity), there is no homology of OGFr with classical opioid receptors in terms of nucleotides or amino acids [1]. Antisense experiments with OGFr and continuous blockade of opioid receptors by the potent opioid antagonist naltrexone (NTX) support that the OGF-OGFr axis is a tonically active inhibitory system targeted to cell replication and homeostasis, and is ligand-dependent for function [1]. Immunoelectron and confocal microscopy have shown that OGFr is localized to the outer nuclear envelope, nucleus, and perinuclear cytoplasm [8]. Gene expression [1], and protein expression [1] of OGFr, as well as binding activity [1] have been identified and characterized in pancreatic cancer cell lines revealing the autocrine nature of this growth regulatory axis.

The action of OGF in neoplasias is targeted to DNA synthesis [4] and, in the cases of pancreatic, squamous cell carcinoma of the head and neck (SCCHN), and colon cancer, is directed toward the G_0_/G_1 _interface of the cell cycle [5]. Further study in SCCHN has revealed that the p16 cyclin dependent inhibitory kinase pathway is the target of OGF [9]. However, the total frequency of mutations and of homozygous deletions involving the p16 gene in human pancreatic carcinoma is nearly 80% [10,11]. Since OGF depresses DNA synthesis and subsequent cell/tissue growth in human pancreatic cancer cells *in vitro *and in xenografts transplanted into nude mice [3], and flow cytometry studies indicate that the G_o_/G_1 _of the cell cycle is altered [5], the question arises as to the mechanism of peptide action on the cell cycle in cancers that have a mutation/deletion of p16. The present investigation examined the specific target(s) in the cell cycle for the OGF-OGFr axis in pancreatic cancers that contain a mutation/deletion of p16.

## Results

### OGF inhibits cell proliferation and retards progression through the cell cycle

**Figure 1:**
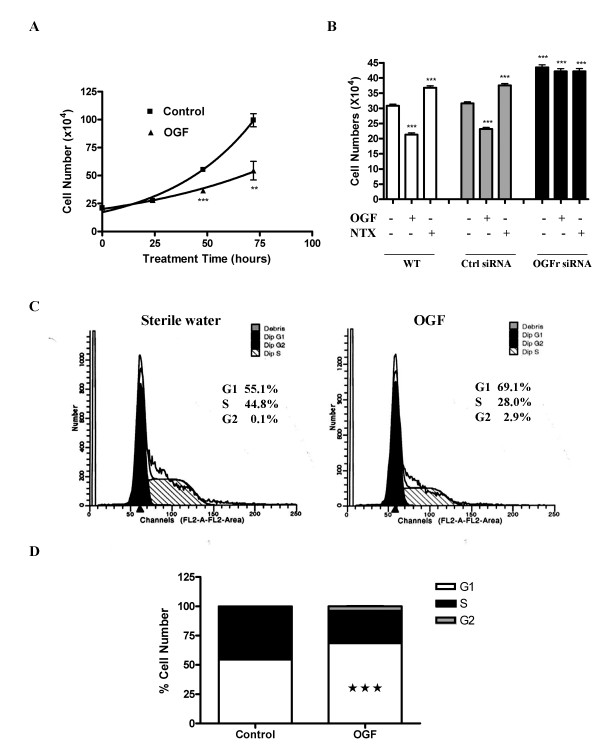
OGF inhibits BxPC-3 growth by arresting cells in G_1_. (A) BxPC-3 cells were grown in the presence of 10^-6 ^M OGF for 72 hours, and counted after 24, 48, or 72 hours of treatment. The number of cells in OGF-treated cultures were significantly reduced from control cultures receiving sterile water at 48 (***p < 0.001) and 72 (**p < 0.01) hours. Cells grown in the presence of the long-acting and potent general opioid antagonist naltrexone (NTX), which blocks the OGF-OGFr axis, enhances cell proliferation. (B) OGFr is required for OGF action on growth. BxPC-3 cells were transfected with OGFr siRNAs or control siRNAs for 24 hours in the presence of 10^-6 ^M OGF, 10^-6 ^M NTX, or sterile water. Cells were harvested at 96 hours and counted with a hemacytometer. Data represent means ± SE for 2 independent experiments. Cell numbers differed from wt or Ctrl siRNA cultures treated with sterile water at p < 0.001 (***). (C) Flow cytometry of synchronized BxPC-3 cells subjected to sterile water (control) or OGF for 21 hours as determined by FACS analysis. (D) Mean percentage of cells in G_1_, S, and G_2 _phases. The percentage of OGF-treated cells in G_1 _was significantly elevated from control values at ***p < 0.001.

Continuous exposure to exogenous OGF inhibited the growth of BxPC-3 human pancreatic cancer cells. At 48 and 72 hours, cell number in the OGF-treated wells was 66% and 55%, respectively, compared to control cultures receiving sterile water (Fig. 1A). Linear regression analysis of the data revealed mean doubling times for the OGF and control groups of approximately 51.0 and 28.6 hours, respectively; these doubling times differed from each other at p < 0.01.

To examine the specificity of OGF for OGFr, knockdown experiments with OGFr-siRNA were conducted (Fig. 1B). Exposure to 10^-6 ^M OGF depressed the growth of wt and control siRNA-treated cells by 31% and 27%, respectively, whereas 10^-6 ^M NTX increased the number of both wt and control siRNA-exposed cells by 19%. BxPC-3 cells subjected to OGFr-siRNA had approximately 41% more cells than wt cultures, and 38% more cells than control siRNA-treated cultures. In contrast to cells expressing OGFr, exposure to 10^-6 ^M OGF or NTX had no further effects on the OGFr-siRNA cultures.

Based on growth curves, the effect of OGF on cell cycle distribution by flow cytometry was analyzed (Fig. 1B). The percentage of OGF-treated cells in the G_0_/G_1 _phase was 69.1% compared to 55.1% of the control cells (Fig. 1C). Correspondingly, the number of OGF-exposed cells in S phase decreased to 28.0% relative to 44.8% of the sterile water control cells (Fig. 1B). The number of cells subjected to OGF in the G_2_/M phase was 2.9% in relationship to 0.1% for control samples.

### OGF treatment does not change total Rb protein but decreases the amount of phosphorylated Rb

**Figure 2:**
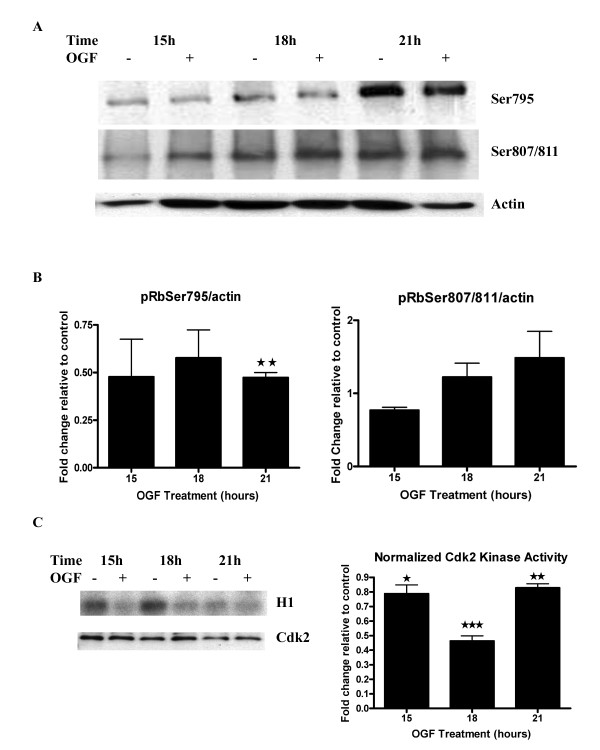
Inhibition of Rb phosphorylation and Cdk2 kinase activity by OGF. (A) Synchronized BxPC-3 cells were grown in the presence of OGF or sterile water and harvested at 15, 18 and 21 hours. Equal amounts of protein were analyzed by Western blot using specific antibodies recognizing phosphorylated Rb (Ser807/811 or Ser795), and normalized to actin. (B) Densitometric analysis of Western blots in (A). Rb phosphorylation after OGF treatment is expressed relative to controls at the respective time point. Cdk2 activity as detected by phosphorylation on Rb Ser795 antibodies was reduced approximately 2-fold in OGF-treated groups relative to controls at all time points, and was significantly repressed at 21 hours (**p < 0.01). The lower level of phosphorylation at early time points resulted in higher variability due to quantifying low signals; as a consequence, the early time points are not statistically significant, but nevertheless the trend is clearly similar. No significant change in Rb phorphorylation related to Cdk4 kinase activity as assessed by phosphorylation at SER807/811 was recorded at any time point. Absolute changes in phosphorylation were less than 48%. (C) Cdk2 kinase activity was evaluated in synchronized BxPC-3 cells treated with OGF for 15, 18 or 21 hours. Cdk2 kinase activity was measured as the capacity of phosphorylation of H1 protein in the presence of radioactive ATP. Densitometric analysis of the kinase assay was performed, and the Cdk2 activity was measured relative to controls. Values represent means ± SE for 3 independent experiments. Kinase activity values from OGF-treated cultures were significantly reduced from control cells at each respective time point (*p < 0.05, **p < 0.01, ***p < 0.001).

The phosphorylation of Rb protein is necessary for cells to progress from G_1 _to the S phase. To elucidate the role of Rb in OGF-induced BxPC-3 cell growth inhibition, Rb expression and the phosphorylated state of Rb were assessed in synchronized BxPC-3 cells. Expression of total Rb protein was not decreased from baseline values after 21 hours of OGF treatment using an antibody at 1:200 dilution. However, the level of phospho-Rb (Ser795), which is specifically phosphorylated by Cdk2/4 in the G_1 _phase [12], was decreased at 15, 18, and 21 hours following exposure to 10^-6 ^M OGF, and reached statistical significance at 21 hours (p < 0.01) (Figs. 2A, B). Although phospho-Rb (pSer807/811) was specifically phosphorylated by Cdk4 in the G1 phase [12], OGF treatment did not alter the results of Western blot analysis of phospho-Rb (pSer807/811) levels (Fig. 2B). These results suggest that Cdk2, not Cdk4, was involved in the OGF-induced cell cycle block at the G_1 _phase in BxPC-3 cells.

### OGF reduces Cdk2 kinase activity

To verify whether the OGF-induced downregulation of pRb was associated with changes in Cdk2, Cdk2 expression and activity levels were determined. The expression of Cdk2 protein did not change following OGF exposure (Fig. 2C). When H1 was used as substrate in immunoprecipitation experiments performed with antibodies against Cdk2, lysates from cells treated with OGF for 15, 18 and 21 hours showed a significant decrease of 78%, 46% and 82%, respectively, in kinase activities compared to sterile water treated control cultures (Fig. 2C). These results demonstrate that the OGF-mediated decrease of pRb was consistent with the reduction in Cdk2 kinase activities.

### OGF does not affect the cyclin E/Cdk2 complex

To examine whether the OGF-induced downregulation of Cdk2 kinase activity was related to a change in cyclin E expression, homogenates of BxPC-3 cells were subjected to Cdk2 immunoprecipitation. Cyclin E protein levels were assessed by Western blotting. The level of the cyclin E/Cdk2 complex after treatment with OGF revealed no change from control levels [Additional file 1].

### p21 expression is upregulated by OGF in a receptor mediated manner

**Figure 3:**
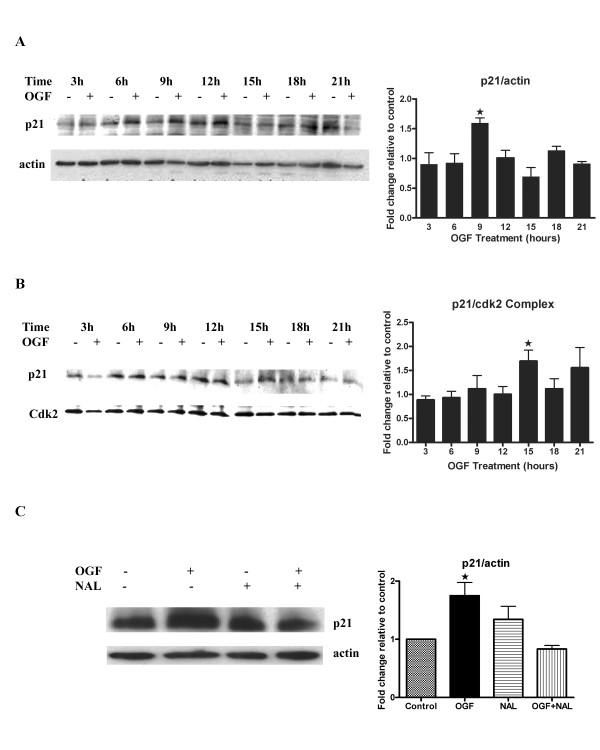
OGF induced p21 expression. (A) BxPC-3 cells were synchronized by nocodazole (67 ng/ml) for 24 hours, and subsequently treated with 10^-6 ^M OGF or sterile water for 3, 6, 9, 12, 15, 18, and 21 hours. Total proteins were resolved by SDS-PAGE, and blotted with p21 specific antibodies. Densitometric analysis of the Western blots was performed, and p21 expression for OGF-treated cells is expressed relative to controls at each time point. The p21 level was significantly (*p < 0.05) elevated from the control group at 9 hours. (B) To examine whether the OGF-induced downregulation of Cdk2 kinase activity was based on p21/Cdk2 complex formation, homogenates of BxPC-3 cells treated with OGF or sterile water were subjected to Cdk2 immunoprecipitation; the resulting proteins were blotted with antibodies to p21. Densitometric analysis of Western blots of immunoprecipitated p21/Cdk2 complexed protein at 3, 6, 9, 12, 15, 18, and 21 hours revealed an increase at 9, 12, 15, 18, and 21 hours, with significant difference between the OGF and 15-hour control group (*p < 0.05). (C) Opioid receptor mediation of OGF action was evaluated in synchronized BxPC-3 cells treated with 10^-6 ^M OGF, 10^-5 ^M naloxone (NAL), both OGF and NAL, or sterile water (Control) for 9 hours. Protein lysates were resolved on SDS-PAGE, and subjected to Western blot analysis for p21 and actin. Densitometric analysis of p21 expression showed that p21 levels of OGF -treated cells were significantly elevated from controls at *p < 0.05; no change was recorded in the NAL and OGF-NAL groups. Data represent means ± SE for 3 independent experiments.

Cell cycle progression depends on both positive and negative regulators. Expression of p21 was evaluated in synchronized BxPC-3 cells after 3, 6, 9, and 12 hours of OGF exposure. p21 was significantly upregulated (p < 0.05) in OGF-treated cells relative to sterile water control cells after 9 hours of drug treatment (Fig. 3A).

Because p21 inhibits Cdk2 by physical interaction, we examined whether the OGF-induced downregulation of Cdk2 kinase activity was based on p21/Cdk2 complex formation. To explore this possibility, homogenates of BxPC-3 cells were subjected to Cdk2 immunoprecipitation, and the precipitated proteins were probed with p21 antibodies. The level of the p21/Cdk2 complex following OGF treatment was elevated at all time points compared to control levels, and reached statistical significance (p < 0.05) at 15 hours (Fig. 3B).

To demonstrate the opioid-receptor mediation of OGF, cells were treated concomitantly with the opioid antagonist naloxone and OGF. OGF-induced upregulation of p21 expression was blocked in cells exposed to both naloxone and OGF; naloxone alone had no effect on p21 expression (Fig. 3C). This result showed that OGF-induced upregulation of p21 expression was receptor mediated. p21 is known as a tumor suppressor gene, functioning as a cell cycle inhibitor by forming heterotrimeric complexes with Cdks and cyclins. Therefore, the data suggest that under the effect of OGF, p21 protein levels were upregulated, and activated the p21-Rb pathway that, in turn, mediated the cell cycle blockade.

**Figure 4:**
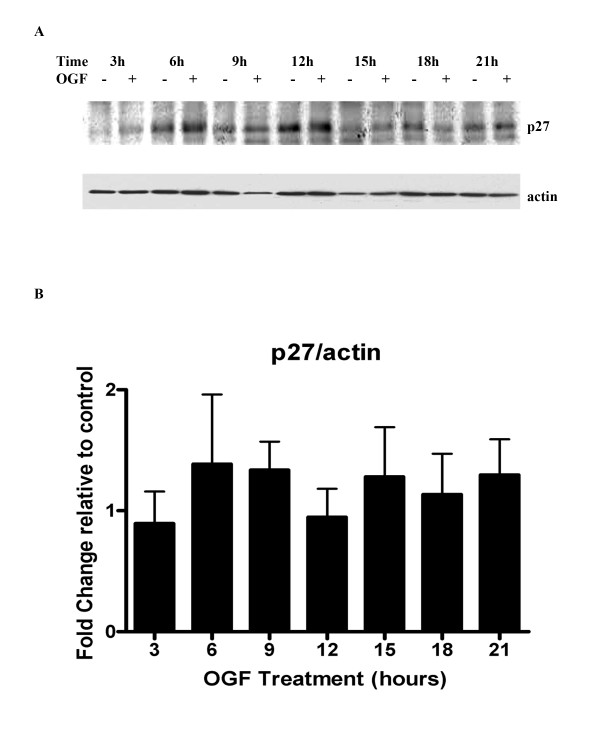
OGF has no effect on CKI p27 expression. BxPC-3 cells were synchronized by nocodazole (67 ng/ml) for 24 hours, and subsequently treated with 10^-6 ^M OGF for 3, 6, 9, 12, 15, 18, or 21 hours. (A) Total protein lysates were resolved by SDS-PAGE and subjected to antibodies specific to p27. (B) Densitometic analysis revealed changes in p27 expression of less than 38% between OGF and sterile water treated values; none of these changes were statistically significant.

Analysis of the expression of the cell cycle inhibitor p27 revealed that OGF treatment had no significant effect on expression levels of this CKI (Fig. 4). p57 protein was not detected on Western blots (1:200 dilution) of BxPC-3 cells. Thus, OGF-treatment results in the induction of only p21 in pancreatic cancer.

### Transfection with siRNAs directed against p21 blocks OGF inhibitory action

**Figure 5:**
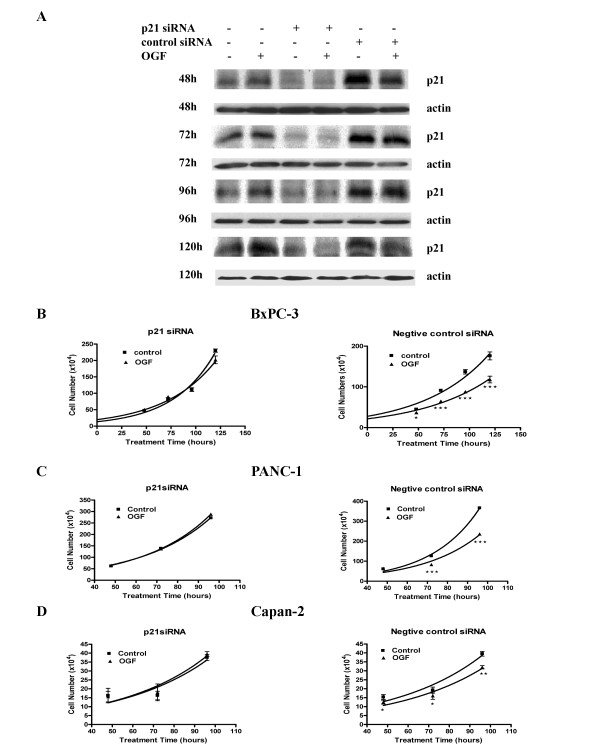
p21 is required for OGF-induced growth inhibition. (A) BxPC-3 cells were transfected with p21 siRNAs or negative control siRNAs for 48, 72, 96, or 120 hours. Total proteins were isolated and separated by SDS-PAGE, and probed with antibodies specific to p21. (B-D) Growth curves for BxPC-3 (B), PANC-1 (C) and Capan-2 (D) cells transfected with either p21 siRNA or negative control siRNA, and grown in the presence or absence of 10^-6 ^M OGF for 96 or 120 hours. Cells were harvested at 48, 72, 96 or 120 hours, and counted with a hemacytometer. Data represent means ± SE for 3 independent experiments. OGF had no effect on cell growth of any cell line transfected with p21 siRNA. Cell numbers for OGF-treated cultures transfected with negative control siRNA were significantly reduced from transfected cells receiving sterile water at *p < 0.05, **p < 0.01, or ***p < 0.001.

To test the role of p21 in OGF-induced inhibitory action on BxPC-3 cell growth, siRNA knockdown experiments were utilized. BxPC-3 cells were treated with p21 siRNA or with negative control siRNA. As revealed by Western blot analysis, the p21 siRNA efficiently reduced the level of p21 protein compared to control cells at 48, 72, 96, and 120 hours (Fig. 5A). Growth analysis of cells transfected with p21 siRNAs and subsequently exposed to OGF for 120 hours, showed that p21 induction is required for the OGF inhibitory action on BxPC-3 cell growth (Fig. 5B). BxPC-3 cells transfected with the negative control siRNAs and exposed to OGF exhibited significant reductions in growth of 17.8%, 28.6%, 35.8%, and 33.0% at 48, 72, 96, and 120 hours, respectively.

To examine the ubiquity of the integral role of p21 as a cyclin-dependent kinase inhibitor, two other human pancreatic cancer cell lines were examined: PANC-1 and Capan-2. In synchronized PANC-1 cells, OGF increased p21 expression at 6 hours relative to control cells. Western blot analysis of protein isolated from these cells transfected with p21 siRNA revealed a 51% reduction in p21 levels in comparison to control cells at 96 hours. Growth curves indicated that OGF had no inhibitory effect on PANC-1 cells lacking p21, but OGF exposure markedly decreased growth by 34.1% at 72 hours and 35.5% at 96 hours in cells transfected with the negative control siRNAs compared to cultures treated with sterile water (Fig. 5C). In Capan-2 cells, OGF increased p21 expression at 3 hours relative to control cultures. Capan-2 cells transfected with p21 siRNA had 60% of p21 relative to control cells at 72 hours. Growth curves revealed that OGF had no inhibitory action on Capan-2 cells lacking p21, but OGF did depress growth of Capan-2 cells transfected with negative control siRNA at 48, 72, and 96 hours (Fig. 5D).

## Discussion

The OGF-OGFr axis has been documented by structural, pharmacological, and biochemical evidence to be present and to function as a regulatory system for growth in human pancreatic cancer. First, OGF and OGFr have been identified in human pancreatic cancer cell lines and tumor tissues by immunohistochemistry [2,3]. Second, OGFr has been characterized in human pancreatic cancer cells and surgical specimens by receptor binding [13,14], and shown to have at least a 10-fold greater affinity for OGFr than any other natural or synthetic opioid peptide (including those specific for μ, δ, or κ classical opioid receptors) [13]. Third, exogenous OGF depresses the proliferation of human pancreatic cancer cells *in vitro *[2,5,6] and in xenografts [3,6], and elimination of endogenous OGF neutralizes this peptide's action on cell replication [2]. However, these previous studies do not directly demonstrate that OGF, which also recognizes other classical opioid receptors [15], also may elicit naloxone-sensitive anti-proliferative signaling through these receptors instead of or in addition to OGFr. The logical extension of this query is that the effects of OGF on the cell cycle may be related to OGFr and/or other opioid receptors. Using OGFr knockdown experiments, the present report now shows, for the first time, that the specific and singular receptor for OGF action on the replication of a human pancreatic cancer cell line is OGFr. Cells with silenced OGFr are not altered by addition of OGF.

Moreover, these cells with a knockdown of OGFr are not influenced by NTX, documenting that other opioid receptors are not involved with the effects of this general opioid receptor antagonist. Thus, the elucidation of the target of OGF in this study is directly – and solely – related to OGFr.

This study shows for the first time that the target of the negative growth regulator, OGF is the cyclin-dependent kinase inhibitor p21. Using a human pancreatic cancer cell line, BxPC-3, that exhibited growth inhibition following exposure to OGF, and flow cytometry observations documenting that OGF impeded cells exiting G_o_/G_1_, we now conclude that the peptide action is related to a key regulator of the G_1 _to S phase transition – the tumor suppressor Rb protein. This would suggest that OGF action in regard to the cell cycle is not broad based, but rather extremely specific to a pathway focused on the phosphorylation of Rb. The effects of OGF on p21 were found to be receptor mediated, as concomitant exposure to the opioid antagonist naloxone blocked the up-regulation of p21 by OGF; naloxone treatment alone had no effect on p21 levels, indicating that there was not simply a counterbalance of up- and down-regulation between OGF and naloxone. Finally, confirmation that p21 was indeed the target of OGF was validated in siRNA studies whereby pancreatic cancer cells exposed to p21 siRNA exhibited no change in growth after exposure to OGF. Thus, our study makes the novel finding that OGF is directed to a singular component of the cell cycle.

This investigation also shows that the action of OGF on the p21 cyclin-dependent inhibitory kinase in pancreatic cancer is not specific to the BxPC-3 cell line, but that the p21-Rb pathway is present and active in a number of other pancreatic cancer cell lines. Thus, two other cell lines exhibiting different levels of differentiation and origin, PANC-1 and Capan-2 cell lines demonstrated that the p21 pathway was targeted by OGF as well. Capan-2 was derived from a well-differentiated adenocarcinoma in the head of the pancreas of a 56-year old male [16], BxPC-3 originated from the body and tail of the pancreas of a 61-year old female and was characterized as a moderately well to poorly differentiated adenocarcinoma [17] whereas PANC-1 was an undifferentiated adenocarcinoma derived from the head of the pancreas from a 56-year old male [18]. Thus, OGF appears to act on the p21 pathway in a variety of human pancreatic cancer cell lines, suggesting that this may be the primary pathway of cell cycle influence of the OGF-OGFr axis in these cancers. Whether OGF also influences p21 *in vivo *in humans is unclear and requires further study.

During the cell cycle progression, Rb is sequentially phosphorylated by different cyclin/Cdk complexes [12]. Different phosphorylation sites in Rb have been demonstrated, with the preferred site being S^807/811 ^by Cdk4/cyclin D and S^795 ^by Cdk2/4 [12]. Our observation that OGF down-regulated the phosphorylation of Rb on S^795 ^is consistent with decreased Cdk2 kinase activity. Cdk2 activity was decreased relative to controls at 15, 18, and 21 hours in response to OGF treatment. This reduction in Cdk2 kinase activity correlated with an increase in Cdk2/p21 complex formation in the OGF-exposed cells relative to control cultures at all time points. We also observed that OGF had no effect on Rb S^807/811 ^phosphorylation, which implies that OGF did not affect Cdk4 activity. Thus, OGF exerts its effects in these cancer cells by inducing Cdk2/p21 complexes.

p21 has been considered as a target for potential anticancer drugs [19,20]. For example, using recombinant adenoviral system (rAd-p21), Joshi *et al.*[19] found the growth of two pancreatic tumor cell lines can be inhibited by p21 *in vitro*, with significant numbers of tumor cells reverting from S to G_o_/G_1_. Studies also showed that TGF-β induced p21^waf1 ^expression in PANC-1 cells, along with subsequent growth inhibition [21]. Our studies in this report suggest that OGF inhibited cell growth by upregulating p21 expression. However, in the case of pancreatic cancer, these changes are transient, indicating that OGF has a cytostatic but not cytotoxic effects. These observations may explain why OGF has no effect on apoptosis and necrosis [22,23].

This study was prompted by gaining an understanding of the nature of OGF's inhibitory influence on the cell cycle. A previous report in SCCHN has shown that OGF is targeted to the p16 cyclin-dependent kinase inhibitory pathway [9]. However, human pancreatic cancers exhibit mutations of the p16 gene in 80% or more of the cases examined [10,11]. The present study utilized human pancreatic tumor cell lines that have mutations of the p16 gene [24] in order to reproduce a model of surgical specimens. The results show that another CKI, p21, is the target of OGF activity. This novel finding indicates that the CKI pathways are malleable to the OGF-OGFr axis, and can accommodate to changes in gene changes. These data also demonstrate that OGF is dependent CKIs to transmit their inhibitory influence on growth. Whether other CKIs can mediate the effects of OGF in the face of mutations of p16 and p21 remains to be determined. Moreover, the CKIs involved with the modulatory action of the OGF-OGFr system in normal cells require clarification.

The clinical ramifications of our findings merit further discussion. A review of the literature indicates that most pancreatic cancers resected in patients contain p21, hence our selection of pancreatic cancer cell lines that were known to have p21. For example, Biankin *et al.*[25] examined the protein expression of p21 in 125 patients and they found that 79% of the tumors were positive for p21. Ahrendt *et al.*[26] recorded p21 protein expression in 56% of the 90 patients with pancreatic cancer, and reported a longer survival in these patients taking chemotherapy [26]. Therefore, neoplasias with the p21 pathway would be predicted to react positively to OGF therapy. Whether they use a different cyclin-dependent inhibitory kinase pathway needs to be determined.

## Conclusion

These data are the first to reveal that the target of cell proliferative inhibitory action of OGF in human pancreatic cancer is a p21 CKI pathway, expanding strategies for diagnosis and treatment of these neoplasias

## Methods

### Cell culture and OGF treatment

Human pancreatic cancer cell lines BxPC-3, PANC-1, and Capan-2 were obtained from the American Type Culture Collection (Manassas, VA), and cultured in RPMI, Delbecco's Modified Medium, or McCoy's 5A medium, respectively. All media was supplemented with 10% FBS, 1.2% sodium bicarbonate, and 0.25% antibiotics (5000 units/ml penicillin, 5 μg/ml streptomycin and 10 μg/ml neomycin).

OGF was purchased from Sigma-Aldrich (St. Louis, MO), dissolved in sterile water, and used at a final concentration of 10^-6 ^M.

### Cell growth and flow cytometry

For growth curves, cells were seeded in 6-well plates at an initial density of ~2 × 10^5 ^cells/well. Fresh media and OGF were added 24 hours after initial seeding, and media and OGF were replaced daily. At appropriate times, the cells were washed with PBS, trypsinized, and viable cell numbers were counted by trypan blue exclusion using a hemacytometer.

For flow cytometry, cells were synchronized with 67 ng/ml nocodazole (Sigma-Aldrich) for 24 hours, followed by three washes with complete media. Cells were released from growth arrest by addition of complete media or OGF-supplemented media. Synchronized cells were treated with 10^-6 ^M OGF for 21 hours; this dosage of OGF was selected because it significantly inhibits cell replication of BxPC-3, PANC-1, Capan-1, and MIA PaCa-2 human pancreatic cells [2] and does not induce apoptosis or necrosis [22] or differentiation [23]. Cells were, harvested with 0.25% trypsin-EDTA (Mediatech, Herndon, VA) and fixed with 70% ethanol at -20°C for up to 7 days before DNA analysis. DNA content was obtained by incubating cells in PBS containing propidium iodide (0.1 mg/ml) and RNase A (0.02 mg/ml) for 15 minutes at 22°C. Fluorescence was measured and analyzed using a BD Biosciences FACScan flow cytometer (San Diego, CA) and Modfit Software (Topsham, ME).

### siRNA knockdown of OGFr

The OGFr-targeted siRNAs (antisense:5'-uagaaacucagguuuggcg-3'; sense: 5'-cgccaaaccugaguuucua-3') were designed and obtained as ready-annealed, purified duplex probes from Ambion (Austin, TX). For transfection, 5 × 10^4 ^cells per well were seeded in 6-well plates containing 1 ml of serum-containing media without antibiotics. In each well, 20 nM OGFr-siRNA or control siRNA solutions in serum-free media were added. Cells were incubated for 4 h at 37°C prior to the addition of OGF. Cultures were incubated an additional 20 h, and then 1 ml fresh complete media either lacking or containing OGF was added. At 96 h cells were collected for computing growth. Two independent experiments were conducted. The control siRNAs were purchased from Ambion.

### Western blot analysis

Synchronized cells (~2 × 10^6^) from each treatment were solubilized in 200 μl RIPA buffer (1X PBS, 10 μM IGEPAL, 1 mg/ml SDS, 5 mg/ml deoxycholic acid), containing protease and phosphatase inhibitors (2 μg/ml aprotinin, 3 mg/ml phenylmethyl sulfonyl fluoride, 1 mM sodium orthovanidate, 1 μM okadaic acid). Total protein concentrations were measured using the DC protein assay kit (Bio-Rad Laboratories, Hercules, CA). Equal amounts of protein (40 μg) were subjected to 10% SDS-PAGE followed by transfer of proteins onto polyvinylidene difluoride (Millipore, Billerica, MA) using standard protocols. The following antibodies were purchased from commercial sources: phospho-Rb (Ser795), phospho-Rb (Ser807/811) (Cell Signaling Technology, Beverly, MA); Cdk2, p57 (Santa Cruz Biotechnology, Santa Cruz, CA); p21, p27, total Rb (BD PharMingen, San Diego, CA); β-actin (Clone AC-15, Sigma-Aldrich). Membranes were probed with secondary anti-rabbit or anti-mouse horseradish peroxidase-conjugated antibodies (GE Healthcare-Amersham Biosciences, Piscataway, NJ), and developed using a chemiluminescence Western blotting detection system.

In order to determine equal loading of total protein samples, blots were reprobed with monoclonal antibody against β-actin at a dilution of 1:2000. If necessary, membranes were processed in stripping buffer (62.5 mM Tris-HCl and 100 mM β-mercaptoethanol/2% SDS, pH 6.7) at 50°C before being reprobed. Means and SE were determined from 3 or more independent experiments.

### Quantitation of Western blots

To quantify expression levels or kinase activity, the optical density of each band was determined by densitometry and analyzed by QuickOne (Bio-Rad Laboratories). Each value was normalized to β-actin from the same blot. To report the changes due to OGF treatment, we calculated the fold increase by dividing the normalized value from the OGF treated samples by the normalized value of control samples at each time point; thus, increases due to OGF have values greater than one, and decreases due to OGF have values less than one.

### Immunoprecipitation and H1 histone kinase assay

For immunoprecipitating protein complexes, cell extracts were prepared as follows: 2 × 10^6 ^cells per sample were rinsed in cold PBS followed by lysis in 200 μl immunoprecipitation buffer (1% NP-40, 10 mM HEPES (pH 7.5), 200 mM NaCl, 5 mM EDTA, 50 mM NaF, 0.2 mM sodium orthovanidate, 1 mM phenylmethyl sulfonyl fluoride,, 2 mM dithiothreitol), 1X Halt™ protease inhibitor cocktail (Pierce, Rockford, IL)). For each immunoprecipitation reaction mixture, a total of 500 μg of protein extract was used. To each sample, 50 μl of protein A beads (Santa Cruz Biotechnology) were added, and incubated at 4°C for 30 min with rotation. Beads were removed by centrifugation at 14000 rpm for 15s. The lysates were then subjected to immunoprecipitation using 10 μl of polyclonal antibody against Cdk2 and incubated at 4°C for 1.5 hours with rotation, followed by addition of 20 μl of protein A beads as the immune complex binding agent. Samples were further incubated at 4°C for 1 hour with rotation. Beads were pelleted by centrifugation at 14000 rpm for 15s, washed three times with 500 μl ice-cold immunoprecipitation wash buffer (1% NP40, 50 mM HEPES, 150 mM NaCl, 1 mM EDTA, 1 mM dithiothreitol), and followed by two washes with ice-cold H1 kinase buffer (50 mM HEPES (pH 7.5), 10 mM MgCl_2_, 10 mM MnCl_2_, 1 mM dithiothreitol). Immunoprecipitates were incubated with 10 μCi γ^32^P-ATP, and 1 μg of histone H1 (Roche) as the substrate for Cdk2. After incubation for 30 min at 30°C, with occasional mixing, the reaction was stopped by the addition of 2× sample loading buffer (Santa Cruz Biotechnology). Proteins were separated by a 12% SDS-PAGE gel, and the Cdk2 kinase activity pattern was visualized by autoradiography of phosphorylated H1.

### p21/Cdk2 assay

Cell extracts were subjected to immunoprecipitation using antibodies against Cdk2 and protein A beads. Immunoprecipitates were separated on a 15% SDS-PAGE gel, and membrane blots probed with p21 antibodies (1:100), and developed using a chemiluminescence Western blotting detection system. Blots were reprobed with Cdk2 antibody (1:200). Three independent experiments were performed.

### SiRNA knockdown of p21^WAF1/CIP1^

The p21-targeted siRNAs were obtained from Santa Cruz Biotechnology, and negative control siRNAs were purchased from Ambion (Ambion, INC., Austin, TX). For transfection, 2 × 10^5 ^cells per well were seeded in 6-well plates containing 1 ml of media without antibiotics. For each well, 1 μl of siRNA stock (20 μM) was mixed with 175 μl of serum-free media. Two μl of Oligofectamine reagent (Invitrogen, Carlsbad, CA) was added to prewashed cells in each well, making the final concentration for siRNA approximately 20 nM. Cells were incubated for 4 hours at 37°C prior to the addition of OGF. Eighteen hours later, 1 ml fresh complete media with or without OGF was added to the cultures; media and OGF were replaced daily. At the indicated time points, cells were collected for growth curves or Western blotting. Three or more independent experiments were conducted.

### Statistical analysis

Values were assessed by one-way analysis of variance (ANOVA) and Newman Keul's post multiple comparison tests.

## Abbreviations

CKI- cyclin-dependent kinase inhibitor; OGF-opioid growth factor; OGFr-opioid growth factor receptor; Rb-retinoblastoma

## Competing interests

The author(s) declare that they have no competing interests.

## Authors' contributions

FC, PJM, MFV, and ISZ participated I the conception and design, analysis, intrepretation of data, drafting of the manuscript, and final approval of the version to be published. FC carried acquisition of the data, and FC and PJM performed the analysis. All authors have read and approved the final manuscript.

## Supplementary Material

Additional file 1 Cyclin E/CDK2 Complex in BxPC3. The data provided document the changes by OGF in regard to the Cyclin E/CDK2 pathway was minimal. Click here for file

## References

[B1] Zagon IS, Verderame MF, McLaughlin PJ (2002). The biology of the opioid growth factor receptor (OGFr). Brain Res Brain Res Rev.

[B2] Zagon IS, Smith JP, McLaughlin PJ (1999). Human pancreatic cancer cell proliferation in tissue culture is tonically inhibited by opioid growth factor. Int J Oncol.

[B3] Zagon IS, Hytrek SD, Smith JP, McLaughlin PJ (1997). Opioid growth factor (OGF) inhibits human pancreatic cancer transplanted into nude mice. Cancer Lett.

[B4] Zagon IS, Wu Y, McLaughlin PJ (1994). Opioid growth factor inhibits DNA synthesis in mouse tongue epithelium in a circadian rhythm-dependent manner. Am J Physiol.

[B5] Zagon IS, Roesener CD, Verderame MF, Ohlsson-Wilhelm BM, Levin RJ, McLaughlin PJ (2000). Opioid growth factor regulates the cell cycle of human neoplasias. Int J Oncol.

[B6] Zagon IS, Jaglowski JR, Verderame MF, Smith JP, Leure-Dupree AE, McLaughlin PJ (2005). Combination chemotherapy with gemcitabine and biotherapy with opioid growth factor (OGF) enhances the growth inhibition of pancreatic adenocarcinoma. Cancer Chemother Pharmacol.

[B7] Smith JP, Conter RL, Bingaman SI, Harvey HA, Mauger DT, Ahmad M, Demers LM, Stanley WB, McLaughlin PJ, Zagon IS (2004). Treatment of advanced pancreatic cancer with opioid growth factor: phase I. Anticancer Drugs.

[B8] Zagon IS, Ruth TB, Leure-duPree AE, Sassani JW, McLaughlin PJ (2003). Immunoelectron microscopic localization of the opioid growth factor receptor (OGFr) and OGF in the cornea. Brain Res.

[B9] Cheng F, Zagon IS, Verderame MF, McLaughlin PJ (2007). The opioid growth factor (OGF)-OGF receptor axis uses the p16 pathway to inhibit head and neck cancer. Cancer Res.

[B10] Cowgill SM, Muscarella P (2003). The genetics of pancreatic cancer. Am J Surg.

[B11] Sakorafas GH, Tsiotou AG, Tsiotos GG (2000). Molecular biology of pancreatic cancer; oncogenes, tumour suppressor genes, growth factors, and their receptors from a clinical perspective. Cancer Treat Rev.

[B12] Zarkowska T, Mittnacht S (1997). Differential phosphorylation of the retinoblastoma protein by G1/S cyclin-dependent kinases. J Biol Chem.

[B13] Zagon IS, Smith JP, Conter R, McLaughlin PJ (2000). Identification and characterization of opioid growth factor receptor in human pancreatic adenocarcinoma. Int J Mol Med.

[B14] Zagon IS, McLaughlin PJ (2006). Opioid growth factor receptor is unaltered with the progression of human pancreatic and colon cancers. Int J Oncol.

[B15] Leslie FM (1987). Methods used for the study of opioid receptors. Pharmacol Rev.

[B16] Kyriazis AA, Kyriazis AP, Sternberg CN, Sloane NH, Loveless JD (1986). Morphological, biological, biochemical, and karyotypic characteristics of human pancreatic ductal adenocarcinoma Capan-2 in tissue culture and the nude mouse. Cancer Res.

[B17] Tan MH, Nowak NJ, Loor R, Ochi H, Sandberg AA, Lopez C, Pickren JW, Berjian R, Douglass HO, Chu TM (1986). Characterization of a new primary human pancreatic tumor line. Cancer Invest.

[B18] Lieber M, Mazzetta J, Nelson-Rees W, Kaplan M, Todaro G (1975). Establishment of a continuous tumor-cell line (panc-1) from a human carcinoma of the exocrine pancreas. Int J Cancer.

[B19] Joshi US, Dergham ST, Chen YQ, Dugan MC, Crissman JD, Vaitkevicius VK, Sarkar FH (1998). Inhibition of pancreatic tumor cell growth in culture by p21WAF1 recombinant adenovirus. Pancreas.

[B20] Chan I, Lebedeva IV, Su ZZ, Sarkar D, Valerie K, Fisher PB (2007). Progression elevated gene-3 promoter (PEG-Prom) confers cancer cell selectivity to human polynucleotide phosphorylase (hPNPase(old-35))-mediated growth suppression. J Cell Physiol.

[B21] Grau AM, Zhang L, Wang W, Ruan S, Evans DB, Abbruzzese JL, Zhang W, Chiao PJ (1997). Induction of p21waf1 expression and growth inhibition by transforming growth factor beta involve the tumor suppressor gene DPC4 in human pancreatic adenocarcinoma cells. Cancer Res.

[B22] Zagon IS, McLaughlin PJ (2003). Opioids and the apoptotic pathway in human cancer cells. Neuropeptides.

[B23] Zagon IS, McLaughlin PJ (2005). Opioids and differentiation in human cancer cells. Neuropeptides.

[B24] Sipos B, Moser S, Kalthoff H, Torok V, Lohr M, Kloppel G (2003). A comprehensive characterization of pancreatic ductal carcinoma cell lines: towards the establishment of an in vitro research platform. Virchows Arch.

[B25] Biankin AV, Morey AL, Lee CS, Kench JG, Biankin SA, Hook HC, Head DR, Hugh TB, Sutherland RL, Henshall SM (2002). DPC4/Smad4 expression and outcome in pancreatic ductal adenocarcinoma. J Clin Oncol.

[B26] Ahrendt SA, Brown HM, Komorowski RA, Zhu YR, Wilson SD, Erickson BA, Ritch PS, Pitt HA, Demeure MJ (2000). p21WAF1 expression is associated with improved survival after adjuvant chemoradiation for pancreatic cancer. Surgery.

