# Supplementary figures and images for: The OGF-OGFr axis utilizes the p21 pathway to restrict progression of human pancreatic cancer
Source: Mol Cancer. 2008 Jan 11;7:5. doi: 10.1186/1476-4598-7-5 (PMC2253554; doi:10.1186/1476-4598-7-5)

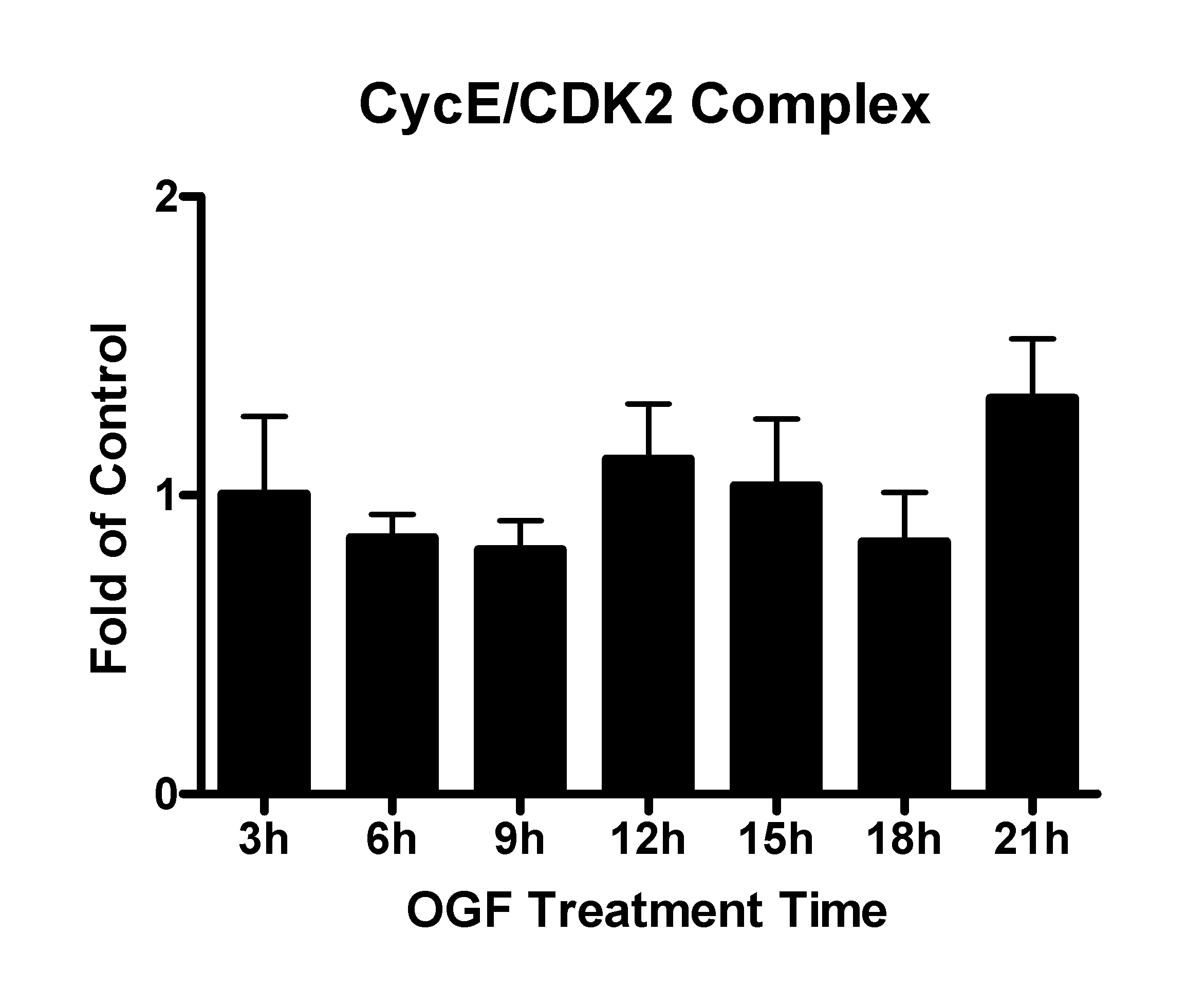

Supplement: Additional file 1 — Cyclin E/CDK2 Complex in BxPC3. The data provided document the changes by OGF in regard to the Cyclin E/CDK2 pathway was minimal. [file 1476-4598-7-5-S1.TIFF]
